# Quantum chemical elucidation of a sevenfold symmetric bacterial antenna complex

**DOI:** 10.1007/s11120-022-00925-8

**Published:** 2022-06-08

**Authors:** Lorenzo Cupellini, Pu Qian, Tu C. Nguyen-Phan, Alastair T. Gardiner, Richard J. Cogdell

**Affiliations:** 1 Department of Chemistry and Industrial Chemistry, University of Pisa, 56124 Pisa, Italy; 2 Materials and Structure Analysis, Thermofisher Scientific, Achtseweg Nordic 5, 5651 GTC Eindhoven, The Netherlands; 3 Institute of Molecular, Cell and Systems Biology, University of Glasgow, Glasgow, G12 8QQ UK; 4 Laboratory of Anoxygenic Phototrophs, Centre Algatech, Novohradská 237 – Opatovický mlýn, 379 01 Třeboň, Czech Republic

**Keywords:** Light-harvesting, Pigment-protein complex, Quantum chemistry, Molecular dynamics, QM/MM, Excitons

## Abstract

The light-harvesting complex 2 (LH2) of purple bacteria is one of the most studied photosynthetic antenna complexes. Its symmetric structure and ring-like bacteriochlorophyll arrangement make it an ideal system for theoreticians and spectroscopists. LH2 complexes from most bacterial species are thought to have eightfold or ninefold symmetry, but recently a sevenfold symmetric LH2 structure from the bacterium *Mch. purpuratum* was solved by Cryo-Electron microscopy. This LH2 also possesses unique near-infrared absorption and circular dichroism (CD) spectral properties. Here we use an atomistic strategy to elucidate the spectral properties of *Mch. purpuratum* LH2 and understand the differences with the most commonly studied LH2 from *Rbl. acidophilus*. Our strategy exploits a combination of molecular dynamics simulations, multiscale polarizable quantum mechanics/molecular mechanics calculations, and lineshape simulations. Our calculations reveal that the spectral properties of LH2 complexes are tuned by site energies and exciton couplings, which in turn depend on the structural fluctuations of the bacteriochlorophylls. Our strategy proves effective in reproducing the absorption and CD spectra of the two LH2 complexes, and in uncovering the origin of their differences. This work proves that it is possible to obtain insight into the spectral tuning strategies of purple bacteria by quantitatively simulating the spectral properties of their antenna complexes.

**Supplementary Information:**

The online version contains supplementary material available at 10.1007/s11120-022-00925-8.

## Introduction

The unique photosynthetic apparatus of purple bacteria comprises two types of light harvesting complexes, LH1 and LH2 (Cogdell et al. 2006; Robert et al. 2003). Both contain bacteriochlorophyll a (BChl) and carotenoid pigments, bound to low molecular-weight $\alpha$/$\beta$ apoprotein dimers. The core complex LH1 is closely associated to the reaction center (RC) and surrounded by a number of LH2 peripheral complexes within the photosynthetic membrane. BChl pigments within LH2 are responsible for the absorption of far-red or near-infrared (NIR) light, and subsequently transfer the energy towards LH1 and finally to the RC. The ring-like oligomeric structure of LH2 complexes and the resulting excitonic properties have attracted considerable interest from spectroscopists and theoreticians (Pajusalu et al. 2011; Mirkovic et al. 2017). The spectroscopic properties of LH2 complexes in the NIR are in fact determined by the excitonic interactions among the BChl $\hbox {Q}_{{y}}$ states, as well as by the interactions between the BChls and the protein matrix.

In all known LH2 antenna complexes, BChls are bound through an axial Mg ligand to the $\alpha$ or $\beta$ apoprotein chains of the complex, and arranged in two rings of high symmetry. The first ring contains one BChl per repeating unit, and is referred to as “B800” ring, because it is responsible for the absorption at $\sim$800 nm in most LH2 complexes. The BChls in the B800 ring are oriented roughly parallel to the membrane plane and fairly spaced. The other ring contains two BChl molecules per repeating unit, bound to His residues in $\alpha$ and $\beta$ apoproteins, and oriented perpendicular to the membrane. This ring features closely spaced BChl molecules with strong excitonic interactions. It is responsible for the red-shifted absorption band at $\sim$850 nm in many LH2 complexes, and thus it is commonly called “B850” ring. The BChl bound to the $\alpha$($\beta$) protein chain are usually denoted as $\alpha$BChl ($\beta$BChl).

Depending on the purple bacterial species, the spectroscopic properties of LH2 complexes can vary greatly, both in the position of the longer-wavelength band and in the shape of the circular dichroism (CD) spectrum (Georgakopoulou et al. 2002). Some species can even synthesize different forms of LH2 in response to mutating light conditions (Cogdell et al. 2006). Such variety of spectrosopic properties arises from the atomistic structure of the complex, which is subtly tuned to the benefit of each bacterial species. Until recently, the only available high-resolution structures of LH2 were those from *Rbl. acidophilus* (formerly *Rps. acidophila*) and from *Rsp. molischianum* (Papiz et al. 2003; Koepke et al. 1996), along with the low-light B800-B820 complex of *Rbl. acidophilus* (McLuskey et al. 2001). While LH2 from *Rbl. acidophilus* features a ninefold symmetry, *Rsp. molischianum* LH2 has an octameric structure. Spectral properties of LH2 complexes from various species could be rationalized based on either of these two structures (Georgakopoulou et al. 2002), with only a few outliers. In the last years, another high-resolution nonameric LH2 from *Rba. sphaeroides* (Qian et al. 2021) and an octameric LH2 from *E. haloalkaliphila* (Leiger et al. 2019) were also resolved.

**Fig. 1:**
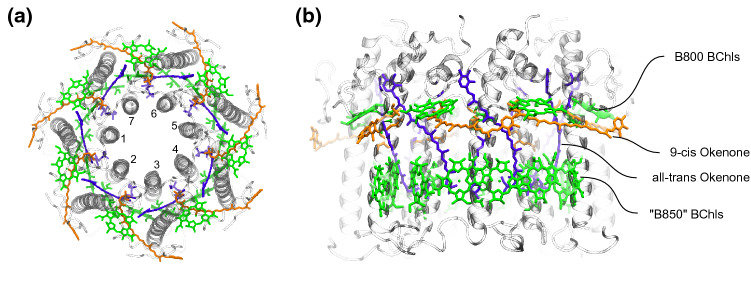
Structure of LH2 from *Mch. purpuratum* [PDB: 6ZXA (Gardiner et al. 2021)]. **a** Top view. The seven repeating units are shown with a number. **b** Side view. All-trans and 9-cis Okenone molecules are shown in purple and orange, respectively; Bacteriochlorophylls are shown in green. The two bacteriochlorophyll rings are labeled B800 and B850 in analogy to the LH2 complex of *Rbl. acidophilus*

Only recently, a new LH2 structure from *Mch. purpuratum* (formerly *Chr. purpuratum*) was resolved by Cryo-Electron microscopy (Cryo-EM) (Gardiner et al. 2021). For the first time, this structure revealed a sevenfold symmetric arrangement of the BChl molecules, which are still arranged in two rings (Fig. 1). It also revealed the binding of two carotenoid molecules (okenone) per repeating unit. Interestingly, *Mch. purpuratum* LH2 also features peculiar spectral properties, different from most LH2 complexes (Georgakopoulou et al. 2002). The long-wavelength “B850” absorption band is blue-shifted at $\sim$830 nm from the usual $\sim$850 nm, and the CD spectrum is unlike any other LH2 complex.

The peculiar oligomeric size of this new LH2 structure sparks additional questions. Namely, does this arrangement affect the exciton band structure of the LH2 complexes? Is the heptameric size related to the blue shift of the B850 band to 830 nm? Answering these questions could provide new insight on how purple bacteria optimize the structure of their peripheral antenna complexes to tune their excitonic and spectral properties.

The spectral properties of LH complexes are modulated by two structural factors (Cogdell et al. 2006; Mirkovic et al. 2017; Curutchet and Mennucci 2017; Cupellini et al. 2020a). Firstly, the distances and orientations among pigments determine the magnitude of exciton couplings, which promote excited-state delocalization, band splitting, and redistribution of dipole strentghs over different states (Jang and Mennucci 2018). Secondly, the identity and structure of the environment surrounding the pigments tunes their site energy by solvatochromic effects, and ultimately results in shifts of the absorption bands (Cupellini et al. 2020a; Renger and Müh 2013). Solvatochromic shifts can be determined both by direct electrostatic tuning of the excitation energies and by indirect effects through the geometry (Cignoni et al. 2022). In LH2 systems, H-bond to the acetyl group of BChls tunes their site energy through the acetyl geometry. In fact, the acetyl group of BChl contributes to the conjugated system when it is in the macrocycle plane. An out-of-plane rotation reduces the conjugation length and thus blue-shifts the BChl ${Q}_y$ site energy (Gudowska-Nowak et al. 1990; Cogdell et al. 2006).

A variety of experimental techniques were employed to investigate exciton delocalization and energy transfer among LH2 BChl rings. Most of these studies were focused on the high-light LH2 complex of *Rbl. acidophilus* (Pajusalu et al. 2011; Kunz et al. 2013; Schlau-Cohen et al. 2013; Kunz et al. 2014; Gellings et al. 2020; Kim et al. 2022) or other octameric or nonameric complexes (Ferretti et al. 2016). Similarly, the availability of high-resolution X-ray structures (Papiz et al. 2003; Koepke et al. 1996) has allowed for quantum chemical investigations of the exciton properties of the BChl rings (Olbrich and Kleinekathöfer 2010; Cupellini et al. 2016; Montemayor et al. 2018; De Vico et al. 2018). On the other hand, studies on *Mch. purpuratum* LH2 have generally focused on the properties of the unusual carotenoid, okenone, and on the carotenoid-to-BChl energy transfer (Polli et al. 2006; Niedzwiedzki et al. 2020). In fact, the unique 7-meric arrangement of LH2 from *Mch. purpuratum* was revealed only very recently (Gardiner et al. 2021).

In this work, we seek to connect the excitonic features of *Mch. purpuratum* LH2 to its structure, using a first-principles protocol to compute the excitonic parameters and finally the spectra (Curutchet and Mennucci 2017; Cupellini et al. 2020a). We employ multiscale polarizable quantum mechanics/molecular mechanics (QM/MMPol) calculations to obtain robust estimations of site energies and excitonic couplings accounting for the surrounding anisotropic protein environment.

Calculations on static structures have been shown to be not representative of the room temperature spectroscopy of LH2 (Cupellini et al. 2016). In addition, such calculations neglect the impact of thermal fluctuations on the exciton structure of the antenna complex. Here we use a strategy based on molecular dynamics (MD) simulations, which allows obtaining an ensemble of representative structures for the pigment-protein complex at room temperature (Cignoni et al. 2022). The ensemble of exciton Hamiltonians obtained along the MD will then characterize both the average exciton levels and their inhomogeneous disorder distribution. Finally, a non-Markovian lineshape theory is used to compute the optical spectra of the LH2 complexes including both static and dynamic disorder components (Cupellini et al. 2020b).

We compare the exciton structure of *Mch. purpuratum* LH2 (from now on LH2*purp*) to the more commonly studied LH2 from *Rbl. acidophilus* strain 10500 (LH2*acid*), which we have previously investigated (Cupellini et al. 2016; Cardoso Ramos et al. 2019). We show that our computational strategy can not only describe the properties of LH2*purp*, but can also explain the spectral differences observed with respect to LH2*acid*. Comparison with experimental absorption and CD spectra reveals the strentghs and limitations of our MD-based strategy, and highlights the need for more accurate empirical force fields to describe exciton disorder and band broadening. Overall, our approach proves successful in connecting structural variations among LH2 complexes to their spectral properties.

## Methods

### Structure preparation and molecular dynamics

The recently reported structure of LH2*purp* (Gardiner et al. 2021) (PDB entry: 6ZXA) was used as a starting point for the simulations. Missing hydrogen atoms were added using the tleap module of AmberTools (Case et al. 2018), considering all residues in their standard protonation states, except for the Mg-binding His, which were considered in their $\delta$ protonation state. The complex was subject to energy minimization before insertion in the model membrane.

The molecular dynamics procedure followed closely the protocol of previous work on nonameric LH2 and other pigment-protein complexes (Cardoso Ramos et al. 2019; Cupellini et al. 2016). Molecular dynamics simulations of LH2*purp* were performed in model 1,2-dioleoyl-*sn*-glycero-3-phosphocholine (DOPC) bilayers. The complex was embedded in a pre-equilibrated DOPC membrane with about 800 lipid molecules in total, solvated with a water layer of 40 Å on both sides and at 0.1 M NaCl. Extra $\hbox {Na}^+$ ions were finally added to neutralize the system.

The system was minimized in three steps: firstly, the lipid molecules in contact with the complex were minimized freezing the rest of the system; next, all lipid tails were minimized; finally the entire system was minimized without restraints. The system was heated from 0 to 100 K in 5 ps (in the NVT ensemble) restraining all atoms except the lipid tails with a 10.0 kcal $\hbox {mol}^{-1}$ Å$^{-2}$) harmonic potential, and then up to 300 K for 100 ps in the NPT ensemble, restraining only the protein and cofactors. The box dimensions were equilibrated by a 5 ns NPT simulation, in which the previously setup restraints on the protein and cofactors were released following a geometric progression from 10.0 kcal $\hbox {mol}^{-1}$ Å$^{-2}$ to 0.8 kcal $\hbox {mol}^{-1}$ Å$^{-2}$. Additional 20 ns of NPT equilibration were performed without further constraints. The production simulations were run for 0.5 μs in the NPT ensemble. In all MD simulations, a 2 fs time step was used. Temperature and pressure were controlled by a Langevin thermostat and the anisotropic Monte Carlo barostat, as implemented in Amber18 Case et al. (2018).

Minimizations and MD simulations were performed using Amber18 program employing the *ff14SB* (Maier et al. 2015) force field for protein and *lipid14* (Dickson et al. 2014) for lipids. The parameters for the BChls were taken from the literature (Ceccarelli et al. 2003). The force field for okenone was adapted from the parameters obtained by Prandi et al. (2016) after assigning the missing parameters with the GAFF force field (Wang et al. 2004). In particular, GAFF was used to describe the phenyl ring of okenone.

### Excitonic states and spectra calculations

The excitations of the multichromophoric aggregate were described in an exciton model, as linear combinations of locally excited (LE) ${Q}_{\text {y}}$ states localized on the pigments, augmented with charge-transfer states. The augmented excitonic Hamiltonian for the LH2 systems reads:

1 $$\begin{aligned} \small {\hat{H}}= & {} \sum _i^{N_{p}} \epsilon _i \left| i\right\rangle \left\langle i\right| + \sum _{ij}^{N_{p}} V_{ij} \left| i\right\rangle \left\langle j\right| + \sum _m^{N_\text {CT}} \epsilon ^\text {CT}_m \left| m\right\rangle \left\langle m\right| \nonumber \\&+ \sum _i^{N_{p}} \sum _m^{N_\text {CT}} \Big ( V_{im}^\text {CT} \left| i\right\rangle \left\langle m\right| + h.c. \Big ) \text {,} \end{aligned}$$

where the indices *i* and *j* run on the locally excited states of the $N_{p}$ BChls, $\epsilon _i$ is the excitation energy of the *i*-th BChl and $V_{ij}$ is the electronic coupling between the *i*-th and *j*-th excitations. The index *m* runs on the CT states, $\epsilon _m^\text {CT}$ is the energy of the *m*-th CT state and $V_{im}^\text {CT}$ is the coupling between the *i*-th locally excited state and *m*-th CT state; *h.c.* denotes Hermitian conjugate.

As the charge-transfer interactions are non-negligible only for pigments in van der Waals contact, only those charge-transfer (CT) states between adjacent pigments in the $\alpha \beta$ ring were considered. This model has been successfully employed for the description of nonameric LH2 complexes (Cardoso Ramos et al. 2019; Cupellini et al. 2018; Nottoli et al. 2018). The standard Frenkel exciton Hamiltonian is recovered neglecting the last two terms of the Hamiltonian (1). All excitonic analyses were performed using the EXAT program (Jurinovich et al. 2018).

Linear spectra were modeled in the disordered exciton approach. Disorder realizations of the exciton Hamiltonian were initially sampled from the molecular dynamics configurations. In order to separate static and dynamic disorder, we used a simple approach employed in previous simulations of LH2 (Cupellini et al. 2016; Cardoso Ramos et al. 2019) and other pigment-protein complexes (Sláma et al. 2020). Namely, we assumed that the environment effect on the site energies contributes to the static disorder. We thus corrected the site energies as

2 $$\begin{aligned} E_{i}^{\mathrm{static}} = E_{i}^{\mathrm{env}} - E_{i}^{\mathrm{vac}} + \langle E^{\mathrm{vac}} \rangle \end{aligned}$$

where $E_{i}^{\mathrm{env}}$ and $E_{i}^{\mathrm{vac}}$ are the site energies calculated on frame *i* in the environment and in vacuum, respectively, and $\langle E^{\mathrm{vac}} \rangle$ is the average of the site energies calculated in vacuum. We note that the resulting distribution of $E_{i}^{\mathrm{static}}$ will have the same average as $E^{\mathrm{env}}$, and the same standard deviation as $E_{i}^{\mathrm{env}} - E_{i}^{\mathrm{vac}}$. From symmetry considerations, the average $\langle E^{\mathrm{vac}} \rangle$ was performed over all equivalent BChls. The distribution of exciton couplings was directly taken from the MD replicas. For each configuration in the MD, diagonal disorder realizations were obtained by sampling a Gaussian distribution of site energies centered in $E_{i}^{\mathrm{static}}$. This was deemed necessary to smoothen the spectrum and recover the experimental broadening of the bands in both complexes. Details on the treatment of static disorder are given in Sect. S1 of the Supplementary Information. The parameters for this additional disorder are reported in the Supplementary Information (Table S4 in the Supplementary Information).

Lineshape calculations were performed for both LH2*purp* and LH2*acid* excitons. The Hamiltonians for the latter were taken from the calculations performed by Cardoso Ramos et al. (2019). Homogeneous lineshapes were computed for each realization of the static disorder using the full cumulant expansion (FCE) formalism in the exciton basis (Ma and Cao 2015; Cupellini et al. 2020b). Contrary to the commonly used cumulant expansion approaches based on Redfield or modified Redfield exciton relaxation rates, the FCE formalism includes both non-Markovian and non-secular effects in the lineshape. These effects have been shown to be crucial for accurate lineshape calculations (Dinh and Renger 2015, 2016; Gelzinis et al. 2015). Spectral densities were modeled as a sum of a high-frequency intramolecular part ($J_\mathrm{vib}(\omega )$) and a low-frequency intermolecular part ($J_\mathrm{env}(\omega )$). The high-frequency part for each BChl was taken from previous QM/MM calculations on the LH2*acid* crystal (Segatta et al. 2017). The complete functional form for the spectral density is reported in Sect. S1 of the Supplementary Information. We assume that the spectral densities do not change significantly from one complex to another. The low-frequency part of the spectral density was taken as a free parameter, which was adapted to best describe the broadening of the exciton bands (See Table S4 in the Supplementary Information).

### Site energies and couplings

For each MD replica, we extracted 50 equally spaced frames in the last 100 ns, for a total of 150 frames. On each frame we computed the site energy of the BChl ${Q}_{\text {y}}$ states and the couplings among them. Excited states were described with the polarizable quantum mechanics/molecular mechanics method (QM/MMPol) (Curutchet et al. 2009; Bondanza et al. 2020; Lipparini and Mennucci 2021). In QM/MMPol, each atom of the MM part is endowed with a dipole polarizability, allowing to describe mutual polarization between QM and MM parts. The QM part was described, as in previous work, at the time dependent density functional theory (TD-DFT) B3LYP/6-31+G(d) level. The Wang parameters (Wang et al. 2011) were used for the classical MMPol part. The phytyl tail of the BChl was kept in the classical part, cutting the CAA-CBA bond. A link atom approach was used to cap the dangling atom.

Electronic couplings in pigment-protein systems are composed of two contributions (Cupellini et al. 2019). The first contribution ($V^\mathrm{Coul}$) is simply the Coulomb interaction between the transition densities of the interacting pigments, whereas the second term ($V^{\mathrm{env}}$) originates from the polarizable medium intervening between the two pigments. The Coulomb term of the coupling was computed analytically from the basis-set expansion of the transition density matrix with the implementation described previously (Iozzi et al. 2004; Curutchet et al. 2009). The direct environment term was calculated in the QM/MMPol framework (Curutchet et al. 2009), which allows considering the anisotropic protein environment around the pigments. Coupling values computed with QM/MMPol were used without further scaling.

### Coupling to charge-transfer states

We computed the effect of CT states with the method proposed in our previous works (Nottoli et al. 2018; Cupellini et al. 2018). All couplings within each adjacent BChl dimer are computed with the multi-FED-FCD diabatization scheme devised in our previous work, (Nottoli et al. 2018) which combines the Fragment Excitation Difference (FED) (Hsu et al. 2008) and Fragment Charge Difference (FCD) (Voityuk and Rösch 2002; Yang and Hsu 2013) methods. This method starts from a calculation of the BChl dimer and recovers LE and CT states and couplings among them. The CT energies were corrected *a posteriori* with the corrected Linear Response (cLR) formalism (Caricato et al. 2006; Loco et al. 2016) to account for the state-specific response of the environment.

In order to well describe both LE and CT states, the calculations on the dimers were performed with a tuned ($\omega$ = 0.195) long-range corrected (LC) BLYP functional and the 6-31G(d) basis set. This functional showed good performance for BChl (Higashi et al. 2014) and CT states of LH2 (Cupellini et al. 2018). As the LC-BLYP/6-31G(d) calculations give higher average Qy energies (by 1008 $\hbox {cm}^{-1}$) than the B3LYP/6-31+G(d) level used for the exciton calculations, we subtracted 1008 $\hbox {cm}^{-1}$ from all LC-BLYP/6-31G(d) energies. In this way, we ensure that the CT-Qy differences reflect those calculated at the LC-BLYP/6-31G(d) level (Cardoso Ramos et al. 2019).

## Results and discussion

In the following, we present the MD characterization of the structural ensemble of LH2*purp* and compare it to the more commonly studied LH2*acid*. Next, we analyze the exciton Hamiltonians of LH2*purp*, and trace the difference between LH2*purp* and LH2*acid* back to the structural parameters. Finally, we assess the quality of our calculated Hamiltonians by comparing the simulated room temperature optical spectra with experiments.

### Dynamical ensemble of LH2

In order to determine the room temperature structural ensemble representing LH2*purp*, three independent replicas were performed of the complex in a model membrane. We seek to understand to what extent this structural ensemble is different from the Cryo-EM structure (Gardiner et al. 2021). RMSD plots (Fig. S1) show a significant deviation of the protein backbone from the Cryo-EM structure in all replicas. The largest RMSD values are found for the C-terminal part of the apoprotein chains, and partially for the N-terminal parts, in agreement with the Cryo-EM B-factors (Gardiner et al. 2021). On the contrary, the helices show rather low and stable RMSD values. The B850 ring of pigments shows comparatively low RMSD values, whereas the B800 ring presents higher displacements. This result is in line with the simulations on LH2*acid* (Cardoso Ramos et al. 2019).

We assessed the equilibration of our simulations with two-dimensional RMSD plots (Fig. S2). These plots compare all pairs of structures along the MDs, and reveal conformational changes occurring mainly in the first 300 ns, both in the helices and in the BChl rings. However, the RMSD within the last 100–200 ns is generally lower than the RMSD from the inifial frames, suggesting that a more equilibrated structure is reached in this time frame.

**Fig. 2:**
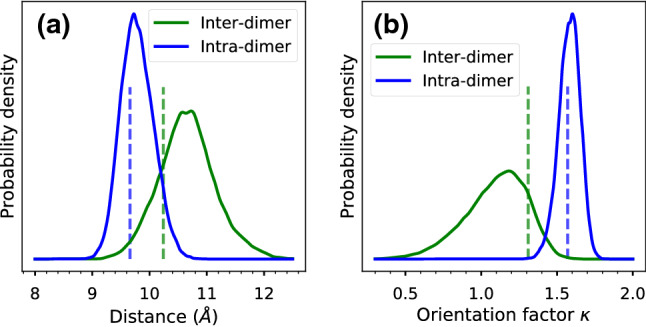
Distribution of **a** center-center distances and **b** orientations between nearest-neighbor BChls in the B850 $\alpha \beta$ ring. “Inter-dimer” refers to the BChl pair bound to the same subunit of $\alpha /\beta$ apoproteins, and “Intra-dimer” refers to the BChl pair of two different apoprotein dimers. The center of each BChl is defined as the midpoint between NB and ND atoms. The orientation factor $\kappa$ is defined as the orientational part of the dipole-dipole interaction, as in Förster theory, between pseudo-dipoles defined on the NB-ND vectors. Vertical lines denote the values measured for the Cryo-EM structure (Gardiner et al. 2021)

The RMSD plots suggest that some reorganization of the pigments occurs when the complex is relaxed at room temperature. Figure 2 shows the distribution of center-center distances and mutual orientations in the closest BChl dimers, compared with the Cryo-EM structure. The pair of BChl molecules within the same $\alpha /\beta$ dimer subunit (Intra-dimer) explores a range of geometries close to the Cryo-EM. A different situation is found for the closest BChl pair in different subunits (Inter-dimer), which during the MD are found at a larger distance, and with a different mutual orientation. The dipolar orientation factor $\kappa$ is significantly smaller in the MD compared to the Cryo-EM, and smaller than the orientation factor of the Intra-dimer BChl pair, which suggests a lowering of the excitonic coupling for the Inter-dimer pair.

**Fig. 3:**
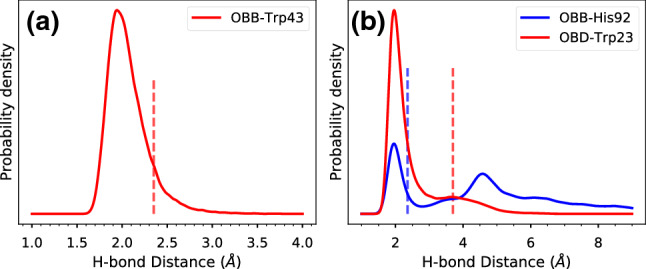
Hydrogen bond interactions between BChls and protein residues in LH2*purp*. **a** Distribution of hydrogen bond distance between the $\beta$-BChl C3$^{1}$ acetyl oxygen and Trp43. **b** Distributions of hydrogen bond distances between the B800-BChl and $\alpha '$-His92 or $\beta$-Trp43. The OBB atom corresponds to the C-13$^1$ carbonyl oxygen, whereas OBD is the C3$^{1}$ acetyl oxygen. Distances are measured between the oxygen atom of the BChl and the polar hydrogen of the residue side chain. Vertical lines denote the values measured for the Cryo-EM structure (Gardiner et al. 2021) after adding and minimizing hydrogens (see “Methods” section)

In LH2*acid*, the Inter-dimer pair is found at a slightly shorter distance than the Intra-dimer pair (Papiz et al. 2003). This finding is also replicated during MD simulations (Cardoso Ramos et al. 2019). Gardiner and co-workers have shown that in LH2*purp* the Inter-dimer pair is more separated (Gardiner et al. 2021). Our MD simulations show that this separation is even larger at room temperature, and also the orientation changes significantly. Thus, although the differences between LH2*acid* and LH2*purp* are noticeable already at the level of Cryo-EM/X-ray structures, only considering thermal fluctuations is it possible to correctly describe the relative arrangements of BChls in the $\alpha \beta$ ring.

Hydrogen bonding plays a primary role in tuning the spectral properties of LH2 complexes. In LH2*acid*, both $\alpha$- and $\beta$-BChls in the B850 ring have their C3$^{1}$ acetyl group H-bonded to protein residues, respectively, Trp45 and Tyr44. Conversely, in LH2*purp*, only the $\beta$-BChl features a H-bond between its C3$^{1}$ acetyl group and the $\beta$-Trp47 in the same polypeptide chain. This hydrogen bond remained stable throughout the three MD replicas. The distribution of H-bond distances (Fig. 3a) is analogous to the $\alpha$-Bchl$\cdots$Trp45 distance in LH2*acid* (Cardoso Ramos et al. 2019), and features longer distances than the $\beta$-BChl$\cdots$Tyr44 counterpart. This finding suggests that the nature of the H-bonding amino acid residue determines the H-bond distance, irrespective of the BChl binding pocket. Interestingly, Jang et al. (2015) analyzed hypothetical LH2 complexes with different symmetry numbers (5–12), and found a similar H-bond between $\beta$-BChl and a Trp in the $\beta$ chain. However, such a hydrogen bond was predicted only for complexes with symmetry number $\ge 8$, and not for a 7-meric complex, for which H-bond to Tyr was predicted. Notably, Trp47 is conserved among LH2 antennas of several species. However, the position of Trp47 is close to the $\alpha \beta$ ring only for LH2*purp*. We conclude that the substitutions in the primary sequence of the $\beta$ polypeptide can affect also the positions of conserved residues and their interaction with the BChls.

In the Cryo-EM structure of LH2*purp*, the B800-BChl interacts with a histidine (His92) of the $\alpha$ chain in the next subunit, through the C3$^{1}$ acetyl oxygen. We investigate the stability of this H-bond in Fig. 3b. Clearly, the C=O$\cdots$His distance reveals two different populations, where the H-bond can be formed or broken. When the H-bond is broken, the C=O$\cdots$His distance can span values ranging from 4 to 8 Å. During our MD simulation, a new hydrogen bond is formed between Trp23 of the $\alpha$ chain and the C13$^1$ carbonyl of B800-BChl. Indeed, Trp23 is mostly found at H-bonding distance with the C13$^1$ carbonyl (red line in Fig. 3b), whereas the conformation found in the Cryo-EM structure has a smaller population. This analysis suggests that the B800-BChl environment is heterogeneous and dynamic. We anticipate that this heterogeneity will impact the energetic disorder of the B800 ring.

### Exciton structure

The exciton structure and optical spectra of the LH2*purp* system are determined by the site energies of the BChls and the couplings among them. It was shown that both factors concur in determining spectral shift between different forms of LH2 (Nottoli et al. 2018). In this Section, we analyze the average site energies and couplings obtained from the MD replicas presented above. We then analyze the exciton states obtained with this average Hamiltonian. In order to uncover the factors underlying the 850 nm to 830 nm shift of the lowest absorption band, we compare the present results with the same values obtained for LH2*acid* (Cardoso Ramos et al. 2019).

The average site energies computed on the MD trajectories of LH2*purp* and LH2*acid* are compared in Table 1. One can immediately note the $\sim$200 $\hbox {cm}^{-1}$ blue shift of $\alpha$-BChl passing from LH2*acid* to LH2*purp*, which is consistent with the loss of one H-bond. Previous calculations (Cardoso Ramos et al. 2019) estimated that a H-bond to the acetyl red shifts the $\hbox {Q}_{{y}}$ excitation by about 140–200 $\hbox {cm}^{-1}$. Therefore, the loss of one H-bond results in a blue shift of the same amount. Interestingly, also the $\beta$-BChl is blue shifted in LH2*purp*, by $\sim$80 $\hbox {cm}^{-1}$, although the $\beta$-BChl features a hydrogen in LH2*purp* as well, which remains stable in our MD simulation (see above). However, the H-bond to Trp found in LH2*purp* is less tight than the H-bond to Tyr found in LH2*acid*. Therefore, the red-shifting effect of a Trp H-bond should be smaller.

**Table 1:** Average site energies of the three distinct BChls along the three MD replicas of LH2*purp*, compared to the ones of LH2*acid* obtained from Cardoso Ramos et al. (2019)

	LH2*purp*	LH2*acid*
$\alpha$-BChl	13,724	13,527
$\beta$-BChl	13,630	13,556
B800-BChl	13,634	13,783

All values are in $\hbox {cm}^{-1}$

Our calculations also reveal that B800-BChl is significantly red-shifted from the value calculated on LH2*acid*. It is difficult to pinpoint the structural changes that cause this shift. In fact, the B800-BChl in LH2*purp* has an opposite orientation with respect to LH2*acid*, in addition to different binding residues. We note anyways that the C13$^1$ carbonyl is surrounded by a nonpolar environment in LH2*acid*, whereas it features a hydrogen bond in LH2*purp*, at least according to our MD simulations (see above).

**Fig. 4:**
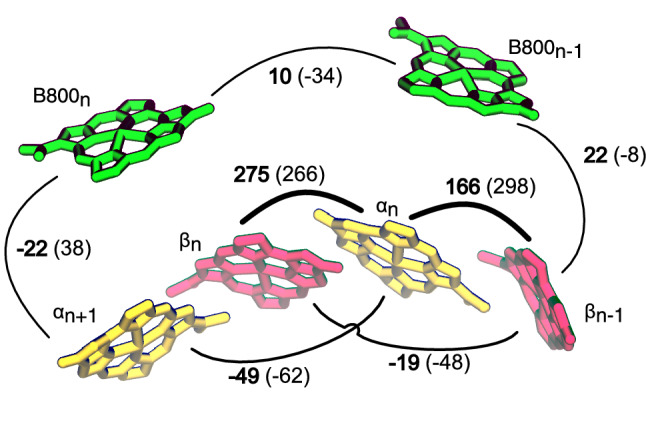
Exciton couplings among the BChls of LH2*purp* in $\hbox {cm}^{-1}$. The values of the same couplings in LH2*acid* are reported in parentheses. All couplings are consistent with an approximate direction NB-ND of the transition dipoles. The labels $\alpha _n$ and $\beta _n$ indicate the $\alpha$ and $\beta$ BChls of the *n*-th repeating unit

The exciton couplings contribute substantially to the band splitting in LH2. Here we calculated couplings along the three MD replicas of LH2*purp* using the multiscale QM/MMPol method, and compared the results with LH2*acid*. The average couplings are compared in Fig. 4. LH2*purp* features generally smaller couplings than LH2*acid*. We observe that the nearest-neighbor couplings reflect the change in mutual orientation and relative distance highlighted in Fig. 2. In fact, the Inter-dimer coupling between close $\alpha$ and $\beta$ BChls belonging to different subunits is the largest coupling in LH2*acid* (298 $\hbox {cm}^{-1}$), but it is almost halved in LH2*purp* (166 $\hbox {cm}^{-1}$). The large difference between the two couplings can be traced back to the increased center-center distance and the smaller orientation factor in this pair (Fig. 2).

In addition to the nearest-neighbor $\alpha \beta$ couplings, also the other couplings show significant deviations from LH2*acid*. Figure 2 reveals that the couplings between $\beta$-Bchls of two consecutive subunits is strongly reduced in LH2*purp*, and also the coupling between $\alpha$-Bchls is slightly smaller. Importantly, we observe that the intra-B800 coupling is reduced by one third passing from LH2*acid* to LH2*purp*. The 10 $\hbox {cm}^{-1}$ coupling obtained on LH2*purp* suggests excitons of the B800 ring will be essentially localized. In addition, we note that inter-ring couplings are still strong, and larger than B800-B800 couplings. Therefore, inter-ring interactions may be important for tuning the optical properties of LH2*purp*.

We are now in a position to analyze the ${Q}_{{y}}$ excitons of LH2*purp* and how they differ from LH2*acid*. To simplify the analysis, we describe the “homogeneous” exciton states obtained from diagonalization of the average exciton Hamiltonian. The spectra calculations in the next Section will instead account for the presence of static disorder in the Hamiltonian parameters. Analyzing the homogeneous exciton states is anyway very useful to understand how site energies and exciton couplings contribute to the exciton energies.

**Fig. 5:**
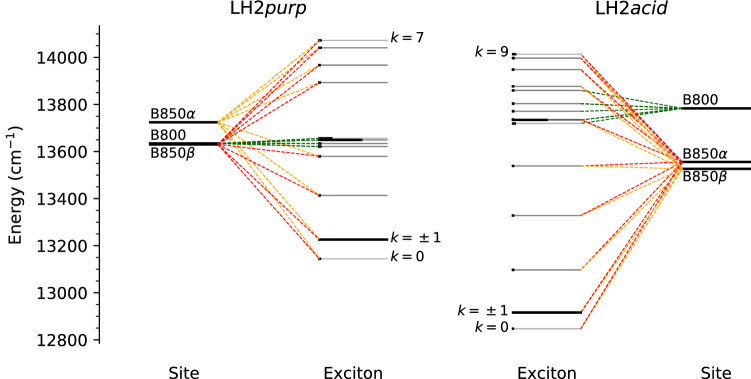
Exciton structure of LH2*purp* (left) and LH2*acid* (right). For each plot, the outermost levels represent the site energies of the BChls, while the innermost ones represent the exciton energies. The length of the solid black bar on the exciton levels represents the relative dipole strength of the transitions. The dashed lines connecting site and exciton states are drawn if the contribution of the site to the exciton is at least 10%. Dashed lines of different colors indicate contributions from different BChls. The labels indicate the bright states ($k=\pm 1$) of the B850 ring and the singly degenerate lowest and highest states of the homogeneous ring

Figure 5 shows the exciton states of LH2*purp* and LH2*acid* and their connection to the ${Q}_{{y}}$ site energies of the BChls. We first focus on the much less coupled B800 ring, which gives rise to a small splitting of the exciton states. This splitting is almost negligible in LH2*purp*, while it is still noticeable in LH2*acid*. Disorder effects are expected to strongly reduce delocalization when exciton couplings are small, although the exciton couplings in the B800 ring of LH2*acid* ($\sim$30 $\hbox {cm}^{-1}$) are probably strong enough to support some delocalization (Novoderezhkin and van Grondelle 2013).

The B850 excitons are split by the strong nearest-neighbor couplings in both LH2 systems. In a homogeneous ring, these states are maximally delocalized and can be assigned on the basis of their symmetry. In the B850 ring, the dipole strength of the $\hbox {Q}_{{y}}$ transitions is almost completely concentrated in the $k = \pm 1$ exciton state, which is the only bright state of this ring. This state is substantially lower in energy in LH2*acid*, even though the site energies are not lowered by the same amount. The exciton band width is also much larger in LH2*acid*, due to the stronger exciton couplings. In addition, the B850 ring of LH2*acid* contains more BChls, which allows more delocalization of the exciton. We expect that this may give rise to a larger band width, and therefore contribute to the lowering of the $k = \pm 1$ state. The exciton band width of LH2 complexes can be experimentally measured by fluorescence anisotropy spectrocopy (Pajusalu et al. 2011). To the best of our knowledge, no such measurements have been attempted for LH2*purp*: these experiments could offer a way to independently validate the exciton couplings calculated here.

**Fig. 6:**
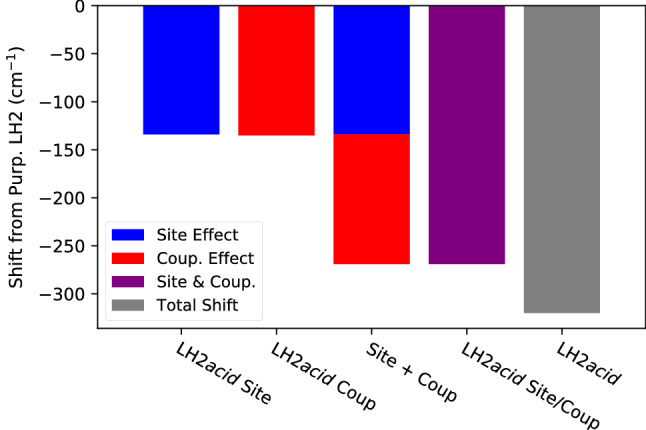
Energy change of the bright “B850” $k = \pm 1$ exciton state for “mixed models” LH2*purp*/LH2*acid*. In these models, some parameters of the LH2*purp* Hamiltonian are changed to their LH2*acid* counterpart (see text). The zero is set to the exciton energy in the LH2*purp* model. The average Hamiltonian is used for this analysis. “Site+Coup” refers to the sum of “Site” and “Coup” contributions

The difference between LH2*acid* and LH2*purp* in the energy of the bright $k = \pm 1$ state arises from a combination of several factors: the site energies are lower in LH2*acid*, the couplings are stronger in LH2*acid*, and finally LH2*acid* features 18 BChls in the B850 ring, compared with the 14 BChls of LH2*purp*. In order to compare these factors, we analyzed some “mixed models”, which we construct by substituting the LH2*purp* excitonic parameters with those of LH2*acid*. The resulting shift of the $k = \pm 1$ bright state is shown in Fig. 6. Moving the $\alpha$-BChl and $\beta$-BChl site energies to the values of LH2*acid* results in a red shift of $\sim$130 $\hbox {cm}^{-1}$. A similar red shift is obtained by substituting all the couplings shown in Fig. 4 with those of LH2*acid*.

If we substitute both site energies and couplings of LH2*purp* with the LH2*acid* counterpart, we obtain a red shift of $\sim$260 $\hbox {cm}^{-1}$. This shows that site energies and couplings have similar and additive effects on the energy of the bright exciton. The obtained red shift is slightly smaller than the difference between LH2*acid* and LH2*purp*, which is $\sim$310 $\hbox {cm}^{-1}$ (grey bar in Fig. 6). This remaining red shift can be imputed to the greater number of pigments in LH2*acid*, which allow more delocalization. Although these results are obtained in the absence of disorder, the trends of Fig. 6 can be expected to qualitatively hold also when static disorder is considered.

Our model predicts a shift of about 310 $\hbox {cm}^{-1}$ between the bright B850 states of LH2*acid* and LH2*purp*. The B850 band of LH2*acid* peaks at 859 nm (Macpherson et al. 2001), whereas LH2*purp* has a maximum at 828 nm (Gardiner et al. 2021). This gives us an estimate of around 440 $\hbox {cm}^{-1}$ for the difference between the bright exciton energies. Our calculations seem to slightly underestimate this difference, as already observed for the high-light and low-light forms of LH2 in *Rbl. acidophilus* (Cardoso Ramos et al. 2019). It is difficult to pinpoint exactly the origin of this discrepancy. One source of error is related to the force field used in MD simulations, which does not describe well the rotation of the C3$^{1}$ acetyl group (Cardoso Ramos et al. 2019).

We note that we have not included the effects of charge transfer states in the previous analysis. Although CT states have an influence on the energy of the LH2 excitons (Nottoli et al. 2018), this influence is strongly modulated by inhomogeneous disorder (Cupellini et al. 2018). For this reason, we will assess the influence of CT states on the spectra in the following section. For the moment, let us briefly analyze the CT energies and couplings, which are reported in Table S1. LH2*purp* features strongly reduced ${Q}_{{y}}$-CT couplings for the Inter-dimer $\alpha \beta$ pair. The Intra-dimer couplings are instead enhanced in LH2*purp*, but this enhancement is smaller in magnitude than the reduction of Inter-dimer couplings. This effect can be again traced back to the arrangement of $\alpha$- and $\beta$- BChls in different subunits of LH2*purp* (see Fig. 2).

### Optical spectra

We assessed the quality of our exciton parameters by simulating the optical spectra for LH2*purp* and LH2*acid*. For each Hamiltonian extracted from the MD simulation, we computed the homogeneous lineshape of the complex (See the “Methods” section). The ensemble lineshape is then obtained by averaging the spectrum over all MD frames. In order to improve the treatment of exciton-vibrational coupling, the lineshape was computed using the full cumulant expansion (FCE) theory (Ma and Cao 2015; Cupellini et al. 2020b). As noted in our previous work, CT states contribute to the broadening of the B850 band. However, it was not possible to match the experimental broadening (Cupellini et al. 2018). Here we used a more practical approach, where we included additional disorder in the exciton Hamiltonian to match the lineshape broadening (The disorder parameters are reported in Table S4), while also including CT states in the exciton Hamiltonian.

**Fig. 7:**
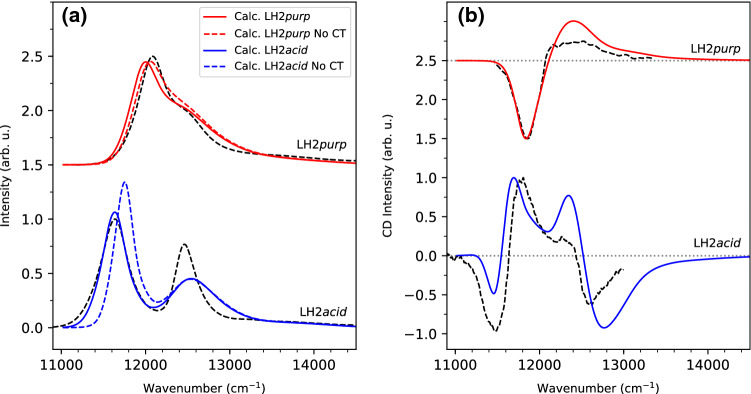
Simulated room temperature optical spectra of LH2*purp* and LH2*acid*, compared with the experiment (black dashed lines). **a** Absorption spectra calculated with the effect of CT states (solid lines) and without the CT effect (dashed lines). Absorption spectra are normalized to the maximum of the band. **b** CD spectra, calculated including CT states. CD spectra are normalized to the larges band (positive or negative). All calculated spectra are identically shifted by − 980 $\hbox {cm}^{-1}$ to account for the error in the QM method. The shift has been adjusted to match the B850 absorption band of LH2*acid*. LH2*purp* spectra are shifted vertically to aid the visual comparison. Experimental spectra of LH2*purp* were taken from Cogdell et al. (1990), while those of LH2*acid* were taken from Cupellini et al. (2016). Spectra are reported on an energy scale to better understand energy differences

The simulated absorption spectra are shown in Fig. 7a (solid lines), along with the experimental counterparts (Cogdell et al. 1990; Cupellini et al. 2016). The calculations reproduce almost quantitatively the blue shift of the B850 band passing from LH2*acid* to LH2*purp*. In addition, the shape of the LH2*purp* absorption well matches the experiment, with a main peak arising from the B850 BChls and a shoulder that can be attributed to B800 BChls. Despite the very good agreement for LH2*purp*, the energy of the B850 band is slightly underestimated by our model, whereas the position of the B800 band is better described. This discrepancy can be traced back, as explained in the previous section, to the incorrect force field description of the C3$^1$ acetyl torsion in the MD simulations. Nonetheless, the error in the peak position is only $\sim$90 $\hbox {cm}^{-1}$, which confirms the good overall accuracy of our calculations.

We can assess the impact of CT states on the LH2 spectra by comparing our results with the calculations that exclude CT states (dashed color lines in Fig. 7a). Clearly, CT states contribute substantially to the B850 band shift in LH2*acid*, and the gap between B850 and B800 bands is only reproduced by the calculations that include CT states. Conversely, the effect on LH2*purp* is less pronounced. As explained above, the inter-dimer ${Q}_{{y}}$-CT couplings are strongly reduced in LH2*purp*, although the intra-dimer ones are enhanced. On average, the reduction of inter-dimer couplings is more substantial, and dampens the effect of CT states in LH2*purp*. We note that the shift between the B850 bands of LH2*acid* and LH2*purp* is reduced when CT states are not included in the calculation. As such, CT states have an impact on the difference between LH2*acid* and LH2*purp*, as previously suggested for the high-light form of *Rbl. acidophilus* LH2 (Nottoli et al. 2018).

Let us now analyze the energetic disorder and band broadening in both LH2 systems. It was necessary to include additional diagonal disorder (See Sect. S1 in the Supplementary Information) to reproduce the width of the B850 band, which means that the B850 energetic disorder is underestimated by MD-based simulations. Without this additional disorder, the B850 band of both complexes is too narrow, and lacks the low-energy tail (See Fig. S4). It has been shown that including CT states is needed to obtain broadened lineshapes, but it is not sufficient (Cupellini et al. 2018). Here, the standard deviation of the environmental disorder for the $\alpha$- and $\beta$- BChls of both complexes (Table S5) is smaller compared to the additional disorder introduced in the simulations, suggesting that other significant sources of disorder must be present in the two complexes. We can put forward several hypotheses in this respect. As suggested in Cupellini et al. (2018), the structural fluctuations experienced by LH2 complexes might occur on much longer timescales than those accessible by MD simulations. Here we used much longer simulations (0.5 μs per replica) than in Cupellini et al. (2018); however LH complexes showed fluctuations in the order of milliseconds, to which our MD is completely blind. Another source of error might be lying in the MM force field description of BChl. As mentioned above, the force field does not describe well the acetyl torsion of BChl (Cardoso Ramos et al. 2019), which might lead to underestimating the structural fluctuation of the BChls and therefore also the site energy disorder. As shown in Fig. S5, the acetyl group is more planar in the MD structure of $\beta$-BChl than in the Cryo-EM structure.

The B800 band of LH2*acid* shows an opposite problem to the B850 band, i.e. it is too broad in our simulation. Apparently, the B800 disorder is exaggerated in our MD simulation, contrary to the B850 one. Indeed, the environmental disorder of B800-BChls obtained from the MD is much larger than the B850 one (Table S5). This can be rationalized by the fact that the B800 BChls have a completely different environment, much more polar than the B850 BChls, and more disordered. The B800 BChls are also in contact with water, as they are outside of the transmembrane region of the protein. The disorder arising from the polar environment seems to be overestimated by our MD simulation. This may be due to deficiencies in the force field description of intermolecular BChl-protein and BChl-water interactions.

It is worth assessing the importance of a MD-based description for LH2*purp*. To this end, we compare the MD-calculated spectra with a static description based on the Cryo-EM structure where the BChls were optimized in a QM/MM scheme (See Sect. S2 in the Supplementary Information for details). The spectrum calculated directly on the Cryo-EM structure is compared with the MD one in Fig. S6. Contrarily to the MD-based spectrum, the Cryo-EM one features only a single band, and does not present the B800 shoulder. The absolute position of the band is slightly different owing to the different internal structure of the BChls, therefore we focus on the relative position of the two bands. This relative position is incorrectly predicted in the Cryo-EM calculation, where B800 and B850 absorption bands essentially overlap. This feature arises from the relative site energies of B800 and B850 BChls: In fact, while in the MD simulation the B800-BChl has a similar site energy to the $\alpha$- and $\beta$- BChls, in the Cryo-EM calculation the B800-BChl has a much lower site energy than the others. Indeed, it seems that the relative site energies of BChl pigments cannot be reliably predicted from the calculations based on the Cryo-EM structure. This result is not unexpected, as static structures (Cryo-EM and X-ray) often present site energy shifts that are not representative of the thermal ensembles (Cignoni et al. 2022).

To gain a deeper insight into the exciton structure of the LH2 complexes, we finally simulated their CD spectra. CD spectra are much more sensitive to variations in the orientations of transition dipoles and in excitonic couplings. At variance with most LH2 complexes, LH2*purp* shows a peculiar CD spectrum with only one intense negative band (Georgakopoulou et al. 2002; Cogdell et al. 1990). Our simulated CD spectrum (Fig. 7b, top) closely reproduces this feature, presenting an almost perfect agreement with the experiment. The peculiar CD shape of LH2*purp* can be traced back to the stronger inter-ring couplings and weaker intra-B800 couplings discussed in the previous section. Without this interaction, the simulations predict a much narrower CD couplet centered around 12 000 $\hbox {cm}^{-1}$, i.e. in the middle of the B850 band (See Fig. S3 in the Supplementary Information). We further note that the positive band is weaker in the experiment than in our simulation. Indeed, the experimental CD of LH2*purp* is nonconservative, probably as a consequence of the mixing with higher excited states of the BChls or with the bright states of the carotenoids. As in this work we have not considered such states, we can only reproduce the conservative part of the CD shape.

The shape of the LH2*acid* CD spectrum, with its characteristic couplets, is also reproduced by our simulation (Fig. 7b, bottom). However, the CD intensities of LH2*acid* are not perfectly reproduced. This problem was also observed previously (Cupellini et al. 2016), and attributed to the extreme sensitivity of the CD couplets to small changes in the transition dipole angles. In our simulations, the CD spectra are averaged on the ensemble of dipole orientations, site energies, and exciton couplings obtained from molecular dynamics. Therefore, small errors in the structural disorder might be amplified CD spectra. Nonetheless, we stress that the sign and positions of the CD band are correctly reproduced by our simulations, without requiring any empirical parameter specifically tuned for reproducing CD spectra (Georgakopoulou et al. 2002).

## Conclusions

In this work, we have investigated the exciton structure of the heptameric LH2 complex from the purple bacterium *Mch. purpuratum*, LH2*purp*. We have employed a multiscale simulation protocol based on a molecular dynamics description of the membrane-solvated complex and QM/MMPol calculations of the excitonic Hamiltonians. This approach is further refined using a non-Markovian lineshape theory to compute absorption and CD spectra of the complex.

Our results reveal the structural and electronic origin of the spectral features of LH2*purp*, and explain the differences from the more commonly studied LH2 from *Rbl. acidophilus*, LH2*acid*. The blue-shifted B850 absorption band of LH2*purp* arises from changes both in the nearest-neighbor exciton couplings and in the site energies of the B850 BChls. Specifically, the couplings among BChls that belong to neighboring units are substantially reduced, due to the increased distance and less favorable orientation found in the heptameric ring. Our MD simulation suggests that the distances and orientations span a wide distribution that deviates from the Cryo-EM values. An additional contribution to the shift is given by charge-transfer states, which cause a larger red shift in LH2*acid* than in LH2*purp*.

Our combined strategy allows for a robust simulation of the optical spectra of LH2*purp*, reproducing the main features of both absorption and circular dichroism spectra. Specifically, the unique CD spectrum of LH2*purp* and the difference from the LH2*acid* one are well predicted by our simulations.

The success of the present approach originates from the combination of state-of-the-art techniques at all levels of the investigation (Cupellini et al. 2020a; Cignoni et al. 2022). The main pillars of this integrated approach are (i) an extensive sampling of the room temperature structural ensemble of LH2 through MD simulations, (ii) a robust calculation of excitonic parameters, such as site energies, excitonic couplings, and charge-transfer couplings through a multiscale QM/MMPol approach accounting for electrostatic and polarization effects of the environment, and (iii) a simulation of optical spectra through a lineshape theory that correctly describes vibronic coupling and static disorder. While each of these ingredients still shows room for improvement (Cignoni et al. 2022), our results point to the force field for BChl as a possible source of inaccuracies. In fact, the remaining discrepancies between spectroscopy simulations and experiments can be traced back to the incorrect force field description of the acetyl torsion (Cardoso Ramos et al. 2019) and possibly of the intermolecular interactions between BChl and the protein. This aspect merits deeper investigation, and further work is ongoing in our group.

## Supplementary Information

Below is the link to the electronic supplementary material.

Supplementary file1 (PDF 970 kb)
