# Supplementary material for: Quantum chemical elucidation of a sevenfold symmetric bacterial antenna complex
Source: Photosynth Res. 2022 Jun 8;156(1):75–87. doi: 10.1007/s11120-022-00925-8 (PMC10070313; doi:10.1007/s11120-022-00925-8)
Supplement: Supplementary file 1 — Supplementary file1 (PDF 970 kb) [file 11120_2022_925_MOESM1_ESM.pdf]

Supplementary Information: Quantum chemical elucidation of a sevenfold symmetric bacterial antenna complex

Lorenzo Cupellini[1,*], Pu Qian[2], Cam Tu Nguyen-Phan[3], Alastair T. Gardiner[4], and Richard J. Cogdell[3]

[1]Department of Chemistry and Industrial Chemistry, University of Pisa, 56124 Pisa, Italy
[*]Corresponding Author; e-mail: lorenzo.cupellini@unipi.it
[2]Materials and Structure Analysis, Thermofisher Scientific, Achtseweg Nordic 5, 5651 GTC Eindhoven, The Netherlands
[3]Institute of Molecular, Cell and Systems Biology, University of Glasgow, Glasgow G12 8QQ, UK
[4]Laboratory of Anoxygenic Phototrophs, Centre ALGATECH, Novohradska 237 Opatovickymlyn, C7379 01 Trebon, Czech Republic

S1 Details on lineshape calculations

Static disorder The inhomogeneous distribution of diagonal and off-diagonal elements of the Hamiltonian was initially obtained from the MD sampling. The distribution of site energies obtained from the environment shifts as explained in Section 2.2 of the main text, whereas off-diagonal elements were taken directly from the values sampled in the MDs. In this way, we obtained one Hamiltonian for each MD configuration, i.e. 150 configurations in total for LH2*purp*. The same procedure was applied to the 50 MD configurations of LH2*acid* obtained in previous work[1].

This distribution of Hamiltonians gives rise to spectra that are too narrow, especially in the B850 band (See Figure S4). For this reason, we added further disorder on the site energies as follows: At each MD configuration, with its own Hamiltonian, 10 realizations were generated by sampling the diagonal elements of the Hamiltonian from a Gaussian distribution. The mean of the distribution of site energies is taken as the site energy E_i of that particular configuration, whereas the standard deviation was determined empirically and is reported in Table S4. We note that such procedure can only increase the disorder with respect to the distribution obtained from the MD configurations. This procedure was applied to both LH2*acid* and LH2*purp*, with slightly different parameters.

Spectral Densities The spectral densities (SD) of Q_y transitions were modeled as a sum of one overdamped Brownian oscillator and M discrete contributions from high-frequency modes:

$$C_j(\omega) = 2\lambda_{c,j}\frac{\omega\gamma_{c,j}}{\omega^2+\gamma_{c,j}^2} + \sum_{k=0}^{M} S_{k,j}\,\omega_{k,j}\,\frac{\omega\gamma_{k,j}}{(\omega-\omega_{k,j})^2+\gamma_d^2} \tag{S1}$$

where j denotes the different BChl pigments (α,β,B800). The overdamped reorganization energies $\lambda_{c,j}$ are reported in Table S4; γ_c is the damping parameter of the overdamped Brownian oscillator, which was set to 100 cm^{-1} for all spectral densities. The frequencies $\omega_{k,j}$ and the Huang-Rhys factors $S_{k,j}$ were calculated previously for LH2*acid*[2].

S2 Calculations on the Cryo-EM structure

The Cryo-EM structure was prepared as explained in Section 2.1 of the main text, but only hydrogen atoms were minimized, whereas all heavy atoms remained in the Cryo-EM positions. We note that the membrane and water molecules were not included in these calculations. In order to avoid biases coming from the BChl bond lengths, the structure of each non-equivalent BChl was partially optimized using the same procedure as in Ref. 1. Namely, a QM/MM constrained optimization was performed by allowing only the coordinates of the BChl to move, while also freezing all dihedral angles at their Cryo-EM value. In the QM/MM optimizations, the MM part was treated with the same parameters used for the molecular dynamics simulations. Only the macrocycle ring of the BChl was included in the QM part. Optimizations were carried out in the ONIOM scheme[3,4]. The structures of an α-BChl, a β-BChl, and a B800-BChl were optimized separately and re-inserted in the Cryo-EM structure replicating the sevenfold symmetry. Optimized structures were used for computing site energies and couplings as described in Section 2.3 of the main text. The site energies and nearest-neighbor couplings obtained on the Cryo-EM structure are reported in Table S6 and compared to the MD averages.

S3 Supplementary Figures

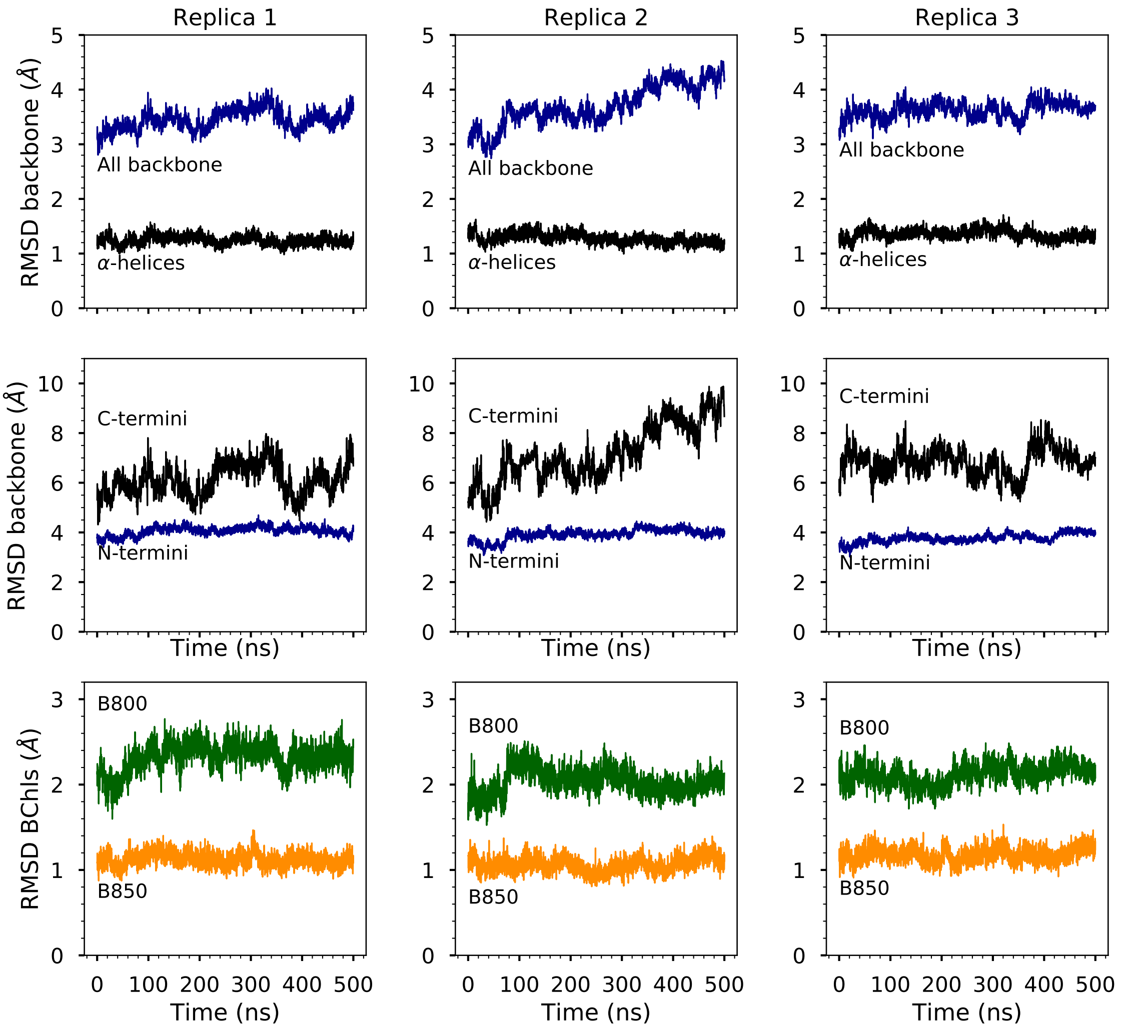

__Figure S1:__ RMSD of the three MD trajectories calculated with respect to the Cryo-EM structure. The RMSD plots of the first two rows were obtained after aligning the entire backbone to the Cryo-EM. The last row refers to each of the B800/B850 BChl rings.

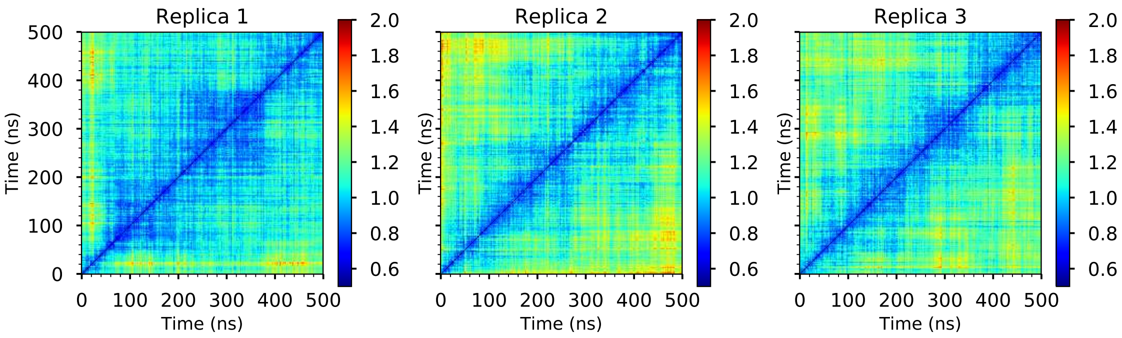

Figure S2: *2D RMSD plots comparing every pair of structures in each MD replica. One structure for each ns was considered in these plots. The color of a point represents the RMSD between backbone structures extracted at different times. Only the transmembrane part of the LH2 apoproteins was considered in this RMSD.*

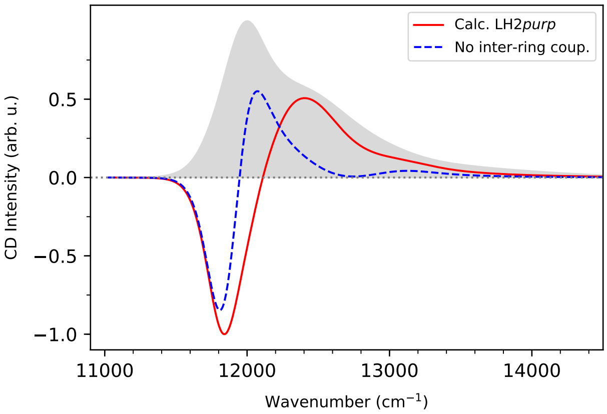

Figure S3: *CD spectrum of LH2purp calculated including (solid red line) and excluding (dashed blue line) the couplings between the two BChl rings. All other parameters are identical. The grey filled curve is the calculated absorption spectrum of LH2purp.*

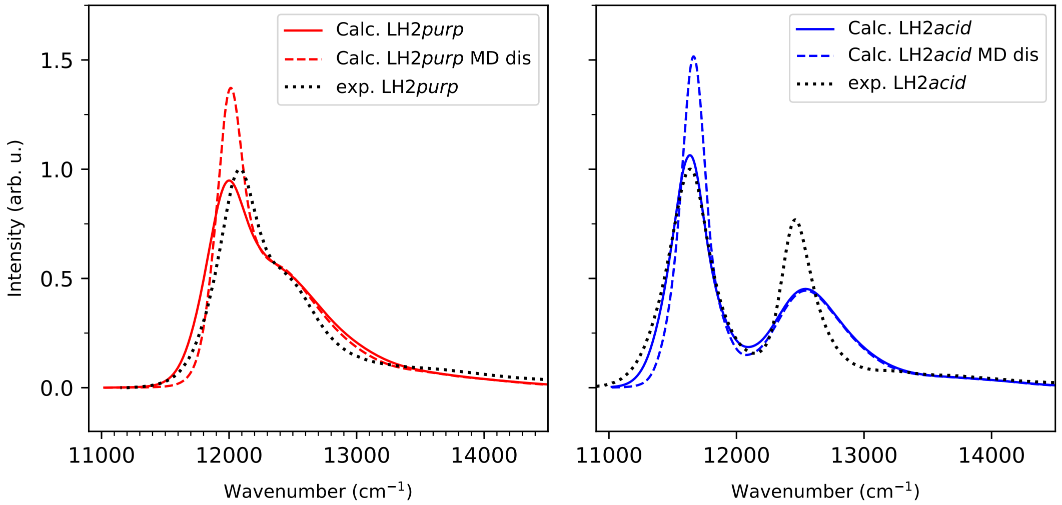

Figure S4: *Comparison between the absorption spectra calculated adding Gaussian diagonal disorder to each MD Hamiltonian (solid lines, equivalent to Figure 7a in the main text) and the same spectra computed without any additional disorder (dashed lines, "MD dis."), i.e. with only the disorder arising from the MD. All spectra have been computed including CT states. All calculated spectra have been shifted by* -980 *cm*$^{-1}$*, and scaled by the same factor.*

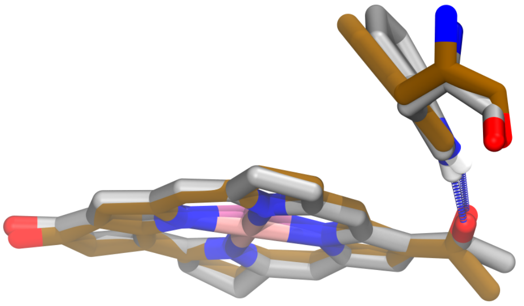

Figure S5: *Structure of the* β*-BChl macrocycle ring as present in the Cryo-EM structure (brown) and in a representative snapshot of the MD simulation (grey). The Trp43 residue H-bonded to the C3*^{1} *acetyl group is also shown.*

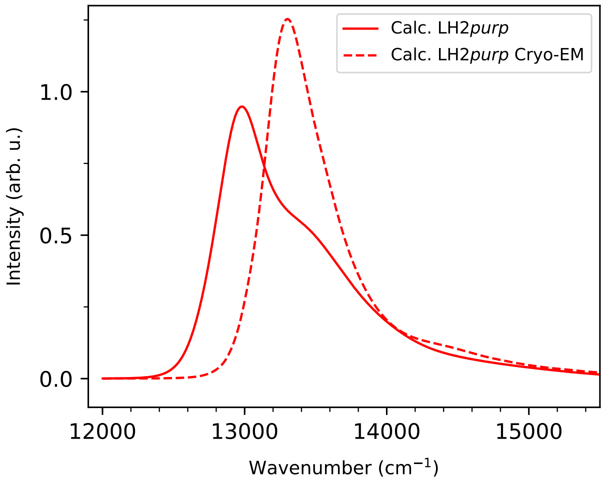

Figure S6: *Comparison between the LH2purp calculated spectrum from the MD simulation and the calculated spectrum from the Cryo-EM structure. Note that no shift was performed on the calculated spectra. Intensities of both spectra were scaled by the same factor.*

S4 Supplementary Tables

Table S1: *Energies of the CT states and couplings between Q_y and CT states of each $\alpha\beta$ dimer, averaged on the MD frames of LH2purp and LH2acid. All values are in cm^{-1}. Note that the CT energies have been corrected for the change of level of theory (see Methods section)*

		LH2*purp*		LH2*acid*	
		CT Energies			
	CT State	Intra-dimer	Inter-dimer	Intra-dimer	Inter-dimer
	$\alpha \to \beta$	19993	24272	22766	20804
	$\beta \to \alpha$	22638	23475	21426	19859
		Q_y / CT Couplings			
Q_y	CT	Intra-dimer	Inter-dimer	Intra-dimer	Inter-dimer
α^*	$\alpha \to \beta$	-423	-51	-170	-400
α^*	$\beta \to \alpha$	-227	-44	-107	-420
β^*	$\alpha \to \beta$	241	35	108	363
β^*	$\beta \to \alpha$	432	68	162	475

Table S2: *Excitonic energies and transition dipoles for LH2purp and LH2acid, computed without the effect of CT states.*

	LH2*purp*		LH2*acid*	
state	E (cm^{-1})	μ (D)	E (cm^{-1})	μ (D)
$k = 0$	13144	2	12847	1
$k = \pm 1$	13226	23	12916	27
$k = \pm 2$	13414	1	13097	0
$k = \pm 3$	13580	1	13328	0
$k = \pm 4$	13893	0	13539	1
$k = \pm 5$	13967	1	13736	1
$k = \pm 6$	14041	3	13877	1
$k = \pm 7$			13948	1
$k = \pm 8$			13997	2
$k = 9$ ($k = 7$)[a]	14072	3	14014	5

[a] For LH2*purp*, the highest exciton state is $k = 7$.

Table S3: *Excitonic energies and transition dipoles for LH2purp and LH2acid, computed with the effect of CT states.*

	LH2*purp*		LH2*acid*	
state	E (cm^{-1})	μ (D)	E (cm^{-1})	μ (D)
$k = 0$	13095	2	12760	1
$k = \pm 1$	13178	23	12830	27
$k = \pm 2$	13368	1	13017	0
$k = \pm 3$	13537	2	13260	0
$k = \pm 4$	13883	0	13495	1
$k = \pm 5$	13958	1	13686	1
$k = \pm 6$	14035	3	13858	1
$k = \pm 7$			13936	1
$k = \pm 8$			13993	2
$k = 9$ ($k = 7$)[a]	14067	3	14013	5

[a] For LH2*purp*, the highest exciton state is $k = 7$.

Table S4: *Disorder parameters for the B800 and B850 pigments of the two considered LH2 systems. We report the standard deviation of the additional inhomogeneous distribution of site energies (σ^{add}) and the reorganization energy for the low-frequency component of the spectral density (λ_c). All quantities are given in cm^{-1}.*

	LH2*purp*		LH2*acid*	
	B850	B800	B850	B800
λ_c	80	160	80	80
σ^{add}	200	140	200	40

Table S5: *Standard deviation σ^{env} of the environmental shifts $E_i^{\text{env}} - E_i^{\text{vac}}$ obtained from the MD simulations. By the correction eq. (2) of the main text, σ^{env} also corresponds to the standard deviation of the diagonal matrix elements in the MD-derived Hamiltonians. All quantities are given in cm^{-1}. Confidence intervals are obtained with bootstrapping.*

	LH2*purp*	LH2*acid*
α-BChl	101 ± 5	85 ± 5
β-BChl	110 ± 6	92 ± 6
B800-BChl	156 ± 10	177 ± 14

Table S6: *Site energies and nearest-neighbor couplings obtained on the cryo-EM structure of LH2purp compared with the MD averages.*

	Cryo-EM (opt)	MD average
α-BChl	14 050	13 724
β-BChl	14 014	13 630
B800-BChl	13 676	13 634
$V_{\alpha\beta}$ (inter)	250	166
$V_{\alpha\beta}$ (intra)	266	275

Supplementary References

[1] F. Cardoso Ramos, M. Nottoli, L. Cupellini, B. Mennucci, Chem. Sci. **2**(42), 9650 (2019). DOI 10.1039/C9SC02886B

[2] F. Segatta, L. Cupellini, S. Jurinovich, S. Mukamel, M. Dapor, S. Taioli, M. Garavelli, B. Mennucci, J. Am. Chem. Soc. **139**(22), 7558 (2017). DOI 10.1021/jacs.7b02130

[3] L.W. Chung, H. Hirao, X. Li, K. Morokuma, Wiley Interdiscip. Rev. Comput. Mol. Sci. **2**(2), 327 (2011). DOI 10.1002/wcms.85

[4] L.W. Chung, W.M.C. Sameera, R. Ramozzi, A.J. Page, M. Hatanaka, G.P. Petrova, T.V. Harris, X. Li, Z. Ke, F. Liu, H.B. Li, L. Ding, K. Morokuma, Chem. Rev. **115**(12), 5678 (2015). DOI 10.1021/cr5004419
